# Robust transcriptional tumor signatures applicable to both formalin-fixed paraffin-embedded and fresh-frozen samples

**DOI:** 10.18632/oncotarget.14257

**Published:** 2016-12-27

**Authors:** Rou Chen, Qingzhou Guan, Jun Cheng, Jun He, Huaping Liu, Hao Cai, Guini Hong, Jiahui Zhang, Na Li, Lu Ao, Zheng Guo

**Affiliations:** ^1^ Key Laboratory of Ministry of Education for Gastrointestinal Cancer, Department of Bioinformatics, Fujian Medical University, Fuzhou 350001, China

**Keywords:** formalin-fixed paraffin-embedded samples, fresh-frozen samples, RNA degradation, gene expression measurements, relative expression orderings

## Abstract

Formalin-fixed paraffin-embedded (FFPE) samples represent a valuable resource for clinical researches. However, FFPE samples are usually considered an unreliable source for gene expression analysis due to the partial RNA degradation. In this study, through comparing gene expression profiles between FFPE samples and paired fresh-frozen (FF) samples for three cancer types, we firstly showed that expression measurements of thousands of genes had at least two-fold change in FFPE samples compared with paired FF samples. Therefore, for a transcriptional signature based on risk scores summarized from the expression levels of the signature genes, the risk score thresholds trained from FFPE (or FF) samples could not be applied to FF (or FFPE) samples. On the other hand, we found that more than 90% of the relative expression orderings (REOs) of gene pairs in the FF samples were maintained in their paired FFPE samples and largely unaffected by the storage time. The result suggested that the REOs of gene pairs were highly robust against partial RNA degradation in FFPE samples. Finally, as a case study, we developed a REOs-based signature to distinguish liver cirrhosis from hepatocellular carcinoma (HCC) using FFPE samples. The signature was validated in four datasets of FFPE samples and eight datasets of FF samples. In conclusion, the valuable FFPE samples can be fully exploited to identify REOs-based diagnostic and prognostic signatures which could be robustly applicable to both FF samples and FFPE samples with degraded RNA.

## INTRODUCTION

The vast majority of clinical tissue samples are routinely fixed in formalin and embedded in paraffin (FFPE) blocks [1–3], and billions of FFPE samples are preserved in hospitals and tissue banks worldwide [4]. Given this wealth of archival clinical specimens from patients with precious clinical and follow-up data [5, 6], the medical research community has strong desire to exploit the FFPE samples to identify transcriptional diagnostic and prognostic biomarkers of tumors. However, FFPE preparation process and storage inevitably degrade RNA [2, 7–9], leading to RNA fragmentation ( up to 50% of which may not contain an intact poly-A tail) [7] and degradation with RIN (RNA Integrity Number) scores usually below three [10, 11]. This problem renders FFPE-isolated nucleic acids unsuitable for gene expression profiling experiments [6, 12] which usually require high-quality fresh-frozen (FF) tissues with RIN score of 6.0 or higher [13–15]. Therefore, FFPE samples are largely limited to immuno-histochemical (IHC) staining and RT-PCR experiments [5, 8, 16–20]. This makes a major limitation for transcriptional analysis when sufficient FF samples are unavailable [1, 21].

Several studies have tried to prove that gene expression profiling can be performed on FFPE samples like FF samples by showing that the gene expression profiles of the FFPE tumor samples are strongly correlated with that of the matched frozen tumor samples [11, 22–28]. However, a high correlation between two gene expression measurements does not guarantee that the two gene expression measurements are close, which will bring uncertainty to the applications of most current disease signatures based on risk scores summarized from expression measurements of the signature genes [29–34]. In this study, through comparing FFPE samples with their paired FF samples, we firstly showed that thousands of genes had at least two-fold change in FFPE samples compared with paired FF samples. Because the expression measurements of the signature genes in FFPE samples cannot be exactly transformed to the expression measurements in FF samples, the type of the risk-scores based signatures determined from FFPE (or FF) samples could not be applied to FF (or FFPE) samples.

Another type of tumor signatures is based on the relative expression orderings (REOs) of genes within samples [35–38], which is highly robust against large measurement variations introduced by experimental batch effects [39–41]. In view of the high correlation between paired FF and FFPE expression profiles [11, 22–28], we reasoned that it would be possible that most of the stable REOs of gene pairs in FF samples could be maintained in the FFPE samples with partial RNA degradation. In this study, we confirmed this reasoning through comparing the REOs in FFPE samples with the REOs in the corresponding paired FF samples obtained from the same patients. Lastly, as a case study to demonstrate the robustness of REOs-based signatures, we developed a REOs-based signature from FFPE samples to distinguishing liver cirrhosis from hepatocellular carcinoma (HCC) and validated this signature in both FF samples and FFPE samples with degraded RNA.

## RESULTS

### The gene expression measurements of FFPE samples affected by RNA degradation

From the The Cancer Genome Atlas (TCGA), we extracted 12, 10 and 5 paired FF and FFPE samples obtained from the same patients with lung adenocarcinoma (LUAD), colon adenocarcinoma (COAD) and breast invasive cancer (BRCA), respectively (Table 1). These paired FF and FFPE samples were used to evaluate the influence of RNA degradation on the gene expression measurements in FFPE samples.

**Table 1:** Description of paired FF and FFPE sample data and normal sample data used in this study

Dataset	Platform	Sample size	Tissue type	Storage type	RIN(FF)	RIN(FFPE)
^#^TCGA_ LUAD	IlluminaHiSeq_RNASeqV2	12 pairs	LUAD	FFPE and FF	8.1~9.4	2.3~2.5
^#^TCGA_ COAD	IlluminaHiSeq_RNASeqV2	10 pairs	COAD	FFPE and FF	7.3~9.8	2.0~2.6
^#^TCGA_ BRCA	IlluminaHiSeq_RNASeqV2	5 pairs	BRCA	FFPE and FF	7.4~9.7	2.1~2.7
GSE54809	GPL6244	7	normal prostate	FFPE	-	-
GSE6956	GPL571	20		FF	-	-
GSE29079	GPL5175	48		FF	-	-
GSE32448	GPL570	40		FF	-	-
GSE46602	GPL570	14		FF	-	-
GSE11682	GPL4133	17		FF	-	-
GSE28204	GPL6480	4		FF	-	-
GSE35988	GPL6480/6848	28		FF	-	-
GSE38241	GPL4133	21		FF	-	-
GSE55597	GPL10558	16		FF	-	-
GSE70768	GPL10558	73		FF	-	-
E-MTAB-2523	IlluminaHiSeq 2000	4	normal liver	FFPE	-	-
GSE41804	GPL570	20		FF	-	-
GSE55092	GPL570	80		FF	-	-
GSE46408	GPL4133	6		FF	-	-
GSE50579	GPL14550	7		FF	-	-
GSE54236	GPL6480	80		FF	-	-
GSE36376	GPL10558	193		FF	-	-
GSE39791	GPL10558	72		FF	-	-
GSE57957	GPL10558	37		FF	-	-

Note:#TCGA_LUAD, #TCGA_COAD and #TCGA_BRCA denote mRNA_seq data of paired FF and FFPE samples for lung adenocarcinoma, colon adenocarcinoma and breast invasive cancer samples from TCGA, respectively.

**Figure 1:**
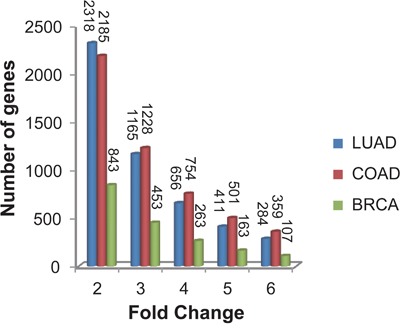
The fluctuation degree of gene expression measurements affected by RNA degradation in FFPE samples Fold changes of DEGs between FFPE samples and their paired FF samples for lung adenocarcinoma (LUAD, shown in blue bar), colon adenocarcinoma (COAD, shown in red bar) and breast invasive cancer samples (BRCA, shown in green bar).

With FDR<0.05, we detected 4133 differentially expressed genes (DEGs) between the 12 FFPE samples and their paired FF samples of LUAD using the Rank Product (RP) algorithm which is resistant to experimental batch effects [42]. Among these DEGs, 2318 genes had at least 2-fold change in the FFPE samples compared with their paired FF samples (Figure 1). Similarly in COAD, we found 4073 DEGs between the 10 FFPE samples and their paired FF samples (RP, FDR<0.05), among which 2185 genes had at least 2-fold change in the FFPE samples compared with their paired FF samples (Figure 1). Similarly in BRCA, we found 1316 DEGs between the 5 FFPE samples and their paired FF samples, among which 843 genes had at least 2-fold change in the FFPE samples compared with their paired FF samples (Figure 1). These results confirmed that gene expression measurements in FFPE samples were widely affected by RNA degradation and expression measurements of thousands of genes had at least 2-fold change in the FFPE samples compared with the FF samples. Therefore, considerable caution must be taken when we interpret gene expression data from FFPE samples.

In addition, the three lists of DEGs for the three types of cancer had 1205 overlaps, among which 99.17% had consistent up- or down-deregulation directions in the FFPE samples compared with the FF samples across the three cancer types (binomial test, *P*-value < 1.0E-16). This result indicated that the genes affected by the RNA degradation were largely independent of the tissue types.

### The robustness of the REOs against RNA degradation in FFPE samples

Using the above FF and FFPE paired samples for LUAD, COAD and BRCA, we evaluated the consistency of REOs of gene pairs between every paired FF sample and FFPE sample extracted from the same patient.

**Figure 2:**
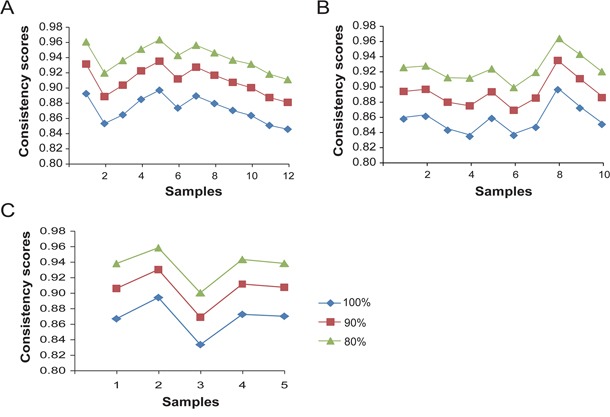
The consistency scores of REOs of gene pairs between every FFPE sample and its paired FF sample The consistency scores between every paired FFPE and FF samples after excluding zero, 10% and 20% gene pairs with the smallest expression differences in each of the FF samples for lung adenocarcinoma **A**., colon adenocarcinoma **B**. and breast invasive cancer **C**.

For all the 200,610,465 gene pairs of measured genes, the average consistency score of the REOs between the FF and paired FFPE samples was 87.22% for LUAD (see Materials and Methods, Figure 2A). It is known that the REOs of gene pairs with small expression differences tend to be unstable due to random measurement variations [43]. After excluding 10% and 20% of the gene pairs with the closest gene expression levels in each of the FF samples, the average consistency scores for the remained gene pairs between the FFPE and paired FF samples increased to 90.96% and 93.96% for LUAD, respectively. Similarly for COAD and BRCA samples, after excluding 10% of the gene pairs with the closest expression levels in the FF samples for each cancer, the average consistency scores for the remained gene pairs between the FFPE and FF samples were larger than 90% and the consistency scores increased as 20% of the gene pairs with the closest expression levels in the FF samples were excluded (Figure 2B and 2C). These results showed that the REOs of gene pairs in FFPE samples were highly robust against RNA degradation.

It has been reported that the yield, purity, and integrity of mRNA progressively decrease with prolonged storage of the paraffin blocks [2, 44, 45]. Here, we evaluated the influence of the storage time on the REOs in the FFPE samples by analyzing seven FFPE normal prostate tissue samples stored for 11~21 years in the GSE54809 dataset. We collected 281 FF normal prostate tissue samples from different data sources (Table 1) and identified 4,008,955 gene pairs with highly stable REOs in at least 99% of these accumulated FF normal prostate tissue samples [38]. Taking them as the golden standard, we found that above 94% of these highly stable REOs were maintained in each of the FFPE normal prostate tissue samples. Similarly, we also analyzed four FFPE normal liver tissue samples stored up to 20 years in the E-MTAB-2523 dataset. Taking 31,429,023 gene pairs with highly stable REOs in at least 99% of 495 FF normal liver tissue samples (Table 1) as the golden standard, we found 92.44% of these REOs were maintained in the FFPE liver tissues stored up to 20 years (Table 2). These results indicated that most of the highly stable REOs of gene pairs in the FF samples remained stable in the FFPE samples although gene expression measurements of FFPE samples were affected by the storage time [2, 44, 45].

**Table 2:** The influence of the storage time on REOs of gene pairs in FFPE samples

Dataset	Storage time(years)	Consistency score
GSE54809	11	0.9668
	12	0.9565
	14	0.9666
	16	0.9464
	18	0.9468
	19	0.9652
	21	0.9611
E-MTAB-2523	0.17	0.9099
	1.08	0.8644
	5.17	0.9757
	20.08	0.9244

Note: The average consistency scores between REOs of FFPE normal samples and stable REOs in normal FF samples.

### A REOs-based signature identified from FFPE samples to distinguish liver cirrhosis from HCC

We collected 82 FFPE samples of liver cirrhosis from the GSE10140 dataset and 80 FFPE samples of HCC from the GSE10141 dataset to train the signature. We extracted 12,567,591 gene pairs with identical REOs in more than 85% of the 82 liver cirrhosis samples, among which we found 143 gene pairs that had the reversal REOs in more than 85% of the 80 HCC samples. From these 143 gene pairs, we selected the five gene pairs (Table 3) with the top-ranked largest geometric mean of the average absolute rank difference in liver cirrhosis and the average absolute rank difference in HCC samples (see Materials and Methods). Using the five gene pairs as the signature, we classified a given sample according to the majority rule: if the REOs of more than half of the five gene pairs in the sample were consistent with the REOs in the liver cirrhosis samples, the sample was identified as the liver cirrhosis; otherwise, the HCC. In the training datasets, 93.75% of the HCC samples and 96.34% of the liver cirrhosis samples were correctly classified. Notably, through literature reviews, we found that CLDN10 [46], CDKN3 [47], CRHBP [48] and NEK2 [49] were reported to be associated with HCC, and SPINK1 [50] was associated with liver cirrhosis.

**Table 3:** The 5-gene-pair signature

Signature	Gene A	Gene B
pair1	CLDN10	SPINK1
pair2	CLDN10	CDKN3
pair3	CLDN10	LCN2
pair4	VIPR1	NEK2
pair5	CRHBP	NEK2

Note: Gene A has a higher expression level than Gene B in liver cirrhosis.

This REOs-based signature was validated in four datasets of FFPE samples and eight datasets of FF samples for liver cirrhosis and HCC. Taking the four datasets of FFPE samples as a whole, 92.57% of the 417 HCC samples and 92.89% of the 225 liver cirrhosis samples were correctly classified. Taking the eight datasets of FF samples as a whole, 94.00% of the 699 HCC samples and 97.11% of the 346 liver cirrhosis samples were correctly classified. As described in Table 4, except the 35 FF samples of HCC in the GSE56140 dataset, above 90% of both the HCC and liver cirrhosis samples in each of the 12 datasets were correctly classified. For the 35 FF samples of HCC in the GSE56140 dataset, seven samples were wrongly classified as liver cirrhosis, possibly due to some unknown factors such as the impurity of the HCC samples that might have no sufficient proportions of tumor cells [51, 52]. In general, this case study demonstrated that a REOs-based transcriptional signature identified from FFPE samples could be applied robustly to both FF and FFPE samples.

**Table 4:** The prediction sensitivity scores of the signature in the validation datasets

Sample type	Dataset	Platform	Number (Sensitivity) of HCC samples	Number (Sensitivity) ofHepatitis/cirrhotic liver samples
FFPE	GSE10142	GPL5474	-	225(0.9289)
	GSE10186	GPL5474	118(0.9068)	-
	GSE19977	GPL8432	164(0.9390)	-
	GSE20017	GPL8432	135(0.9259)	-
FF	GSE63898	GPL13667	228(0.9342)	168(0.9583)
	GSE25097	GPL10687	268(0.9478)	40(1.0000)
	GSE56140	GPL18461	35(0.8000)	34(0.9706)
	GSE36411	GPL10558	42(0.9524)	21(0.9524)
	GSE6764	GPL570	35(0.9429)	13(1.0000)
	GSE9843	GPL570	91(0.9780)	-
	GSE17967	GPL571	-	47(1.0000)
	GSE57725	GPL14951	-	23(0.9565)

## DISCUSSION

To identify transcriptional diagnostic and prognostic biomarkers of tumors, researchers have strong desire to exploit the wealth of FFPE samples preserved in hospitals and tissue banks with precious clinical and follow-up data [5, 6, 53, 54]. However, as shown in this study, the expression measurements of thousands of genes had at least two-fold change in FFPE samples compared with paired FF samples due to the RNA degradation. Therefore, for transcriptional signatures based on risk scores summarized from the expression measurements of the signature genes, risk score thresholds predefined from FFPE (or FF) samples could not be applied to FF (or FFPE) samples directly. The intrinsic problem of incomparable gene expression measurements between FFPE and FF samples cannot be solved even if we could exactly measure low levels of gene expression in FFPE samples by RT-qPCR. In contrast, we found that the vast majority of the REOs of gene pairs in FFPE samples were not affected by RNA degradation. The robustness of REOs against partial RNA degradation makes it possible that REOs-based transcriptional signatures identified from FFPE samples could be applied robustly to both FF and FFPE samples. As demonstrated by the case study, a REOs-based signature consisting of five gene pairs extracted from FFPE samples could be applied to both FF and FFPE samples to distinguish liver cirrhosis from HCC. Thus, the precious FFPE samples could be fully exploited for the identification of REOs-based transcriptional signatures of tumors.

On the other hand, our analyses also showed that the REOs of some gene pairs, especially those gene pairs with small expression differences in FF samples, were not maintained in their paired FFPE samples, indicating that the influence of RNA degradation on some genes’ expression levels in the FFPE samples might be too large to remain their REOs in the FF samples. This result also suggested that subtle quantitative information of gene expression measurements of FFPE samples are unreliable, whereas the seemingly disadvantage of REOs analysis without using some subtle quantitative information of gene expressions is in fact a unique advantage. Especially, we could choose gene pairs with larger expression differences to develop robust REOs-based signatures, excluding gene pairs with small expression differences which tend to be unstable due to random variations of measurement [43]. This strategy would keep sufficient information for prognostic signature detection due to the widely correlated prognostic gene expressions [55]. In general, the subtle quantitative information of gene expression measurements are quite error-prone and uncertain due to various technical artifacts or ‘batch effects’ introduced by the differences in reagent lots, reaction conditions and operators [56–60]. Data normalization methods, such as Combat [61], DWD [57] and XPN [62], could distorts real biological signals [63]. In contrast, the REOs of gene pairs within samples are insensitive to experimental batch effects and data normalizations [64, 65] and thus could provide more accurate and robust patient-specific information for clinical applications [38]. In facts, prognostic signatures based on within-sample REOs have be successfully identified and validated for breast cancer [35, 37, 66, 67], lung cancer [68] and hepatocellular carcinoma [39]. Nevertheless, as shown in this study, that RNA degradation can affect some REOs of genes in FFPE samples in our analyses, it is still necessary to develop new technologies for RNA extraction protocols, RNA amplification and labeling methods to enhance the transcriptome data quality from FFPE samples [25, 28, 69–71]. Especially, because measurement of low levels of gene expression in FFPE samples by RT-qPCR is feasible, it is desirable to develop RT-qPCR kit for translating the REOs-based signatures to clinical applications.

In summary, the REOs-based method will enable gene expression analysis of FFPE samples with RNA degradation that are widely stored in pathology archives around the globe.

## MATERIALS AND METHODS

### Data and preprocessing

All gene expression data analysed in this study were downloaded from the GEO ( http://www.ncbi.nlm.nih.gov/geo/) [72], ArrayExpress ( http://www.ebi.ac.uk/arrayexpress/) [73] and TCGA ( http://cancergenome.nih.gov/), as described in detail in Table 1. For the mRNA-seq profiles of level 3 in TCGA, we removed those genes with zero expression values in both FF and FFPE samples and the remained 20031, 20201 and 20029 genes were analyzed for lung adenocarcinoma, colon adenocarcinoma and breast invasive cancer samples, respectively.

For the data measured by the Affymetrix platform, the Robust Multi-array Average algorithm [74] was used to do background adjustment for the raw mRNA expression data (.CEL files). For the data measured by the Illumina platform, we directly downloaded the processed data. For the data measured by the Agilent platform, we downloaded the raw fluorescent signal intensities data of the channel (gMedianSignal or rMedianSignal) for normal samples and used the intensities to minus the corresponding background signal intensities as the probe-expression matrix. Each probeset ID was mapped to Entrez gene ID with the platform file. If a probeset was mapped to multiple or zero gene, then the data of this probeset was deleted. If multiple probesets were mapped to the same gene, the expression value for the gene was defined as the arithmetic mean of the value of multiple probesets.

### Evaluation of the REOs of gene pairs in each FFPE sample compared with its paired FF sample

All the genes in a sample are ranked according to their expression levels in ascending order. Pairwise comparisons are performed for all genes in each FF sample. Then, we calculated the rank difference for each gene pair in each FF samples by the equations as following:

$$R_{ij}=|R_{i}-R_{j}|$$

R_i_ and R_j_ represent the ranks of gene i and j in FF sample, respectively, and R_ij_ is the absolute rank difference between the two genes. The gene pairs with the smallest R_ij_ were considered to have closest expression levels.

The consistency score of these gene pairs in its paired FFPE sample was calculated as *k/n*, where *n* was the number of the gene pairs in FF samples and *k* was the number of gene pairs with the consistent REOs in the FFPE and FF samples.

### Identification of highly stable REOs in normal tissue

For a particular tissue, pairwise comparisons were performed for all genes to identify gene pairs with stable ordering in accumulated normal samples from different data sources. For each gene pair (Gi, Gj), being viewed as an event with only two possible outcomes (Gi>Gj or Gi<Gj), the gene pairs which the expression level of Gi was higher (or lower) than that of Gj in more than 99% of accumulated normal samples were defined as highly stable gene pairs.

### Developing a REOs-based signature to distinguish liver cirrhosis from HCC

Firstly, a gene pair (Gi and Gj) was selected when its REO, Gi > Gj in expression level, was identical in more than 85% of the liver cirrhosis samples, and was reversed (Gi <Gj) in more than 85% of the HCC samples. After selecting all such reversal gene pairs, we calculated the rank difference for each gene pair in each of the HCC or liver cirrhosis samples.

$$\text{a}vgRij=\sqrt{mean[Rij(cirr)]*mean[Rij(hcc)]}$$

Let *mean*[Rij(cirr)] and *mean*[Rij(hcc)] represent the means of absolute rank differences of the gene pair (i, j) in all liver cirrhosis samples and all HCC samples, respectively. Then, we calculated the geometric mean of the *mean*[Rij(cirr)] and the *mean*[Rij(hcc)] to evaluate the reversal degree of the gene pair. The larger this geometric mean, the larger reversal degree of the REO for the two genes between the liver cirrhosis and HCC samples.

Finally, among all the reversal gene pairs, the gene pairs with the largest geometric mean of the absolute rank differences in liver cirrhosis and HCC samples were selected as the signature. For a given sample, if the REOs of more than half of the gene pairs signature in the sample were consistent with the REOs in the liver cirrhosis sample, the sample was identified as the liver cirrhosis; otherwise, the HCC.

## Abbreviations

FFPE: formalin-fixed paraffin-embedded
FF: fresh-frozen
REOs: relative expression orderings
RIN: RNA Integrity Number
RP: Rank Product
FDR: False Discovery Rate
LUAD: lung adenocarcinoma
COAD: colon adenocarcinoma
BRCA: breast invasive cancer
DEGs: differentially expressed genes
HCC: hepatocellular carcinoma
